# Recognizing emotions in bodies: Vagus nerve stimulation enhances recognition of anger while impairing sadness

**DOI:** 10.3758/s13415-021-00928-3

**Published:** 2021-07-15

**Authors:** Laura Steenbergen, María J. Maraver, Rossana Actis-Grosso, Paola Ricciardelli, Lorenza S. Colzato

**Affiliations:** 1 Cognitive Psychology Unit, Institute of Psychology, Leiden University & Leiden Institute for Brain and Cognition, Leiden, The Netherlands; 2 Clinical Psychology Unit, Institute of Psychology, Leiden University, Wassenaarseweg 52, 2333AK Leiden, the Netherlands; 3 Research Center for Psychological Science (CICPSI), Faculty of Psychology, University of Lisbon, Lisbon, Portugal; 4 Department of Psychology, University of Milano-Bicocca, Milan, Italy; 5 Milan Centre for Neuroscience, Milan, Italy; 6 Cognitive Neurophysiology, Department of Child and Adolescent Psychiatry, Faculty of Medicine, Technical University Dresden, Dresden, Germany; 7 Department of Cognitive Psychology, Institute of Cognitive Neuroscience, Faculty of Psychology, Ruhr University Bochum, Bochum, Germany

**Keywords:** Emotion recognition, Vagus nerve, tVNS, Brain stimulation

## Abstract

According to the Polyvagal theory, the vagus nerve is the key phylogenetic substrate that supports efficient emotion recognition for promoting safety and survival. Previous studies showed that the vagus nerve affects people’s ability to recognize emotions based on eye regions and whole facial images, but not static bodies. The purpose of this study was to verify whether the previously suggested causal link between vagal activity and emotion recognition can be generalized to situations in which emotions must be inferred from images of whole moving bodies. We employed transcutaneous vagus nerve stimulation (tVNS), a noninvasive brain stimulation technique that stimulates the vagus nerve by a mild electrical stimulation to the auricular branch of the vagus, located in the anterior protuberance of the outer ear. In two sessions, participants received active or sham tVNS before and while performing three emotion recognition tasks, aimed at indexing their ability to recognize emotions from static or moving bodily expressions by actors. Active tVNS, compared to sham stimulation, enhanced the recognition of anger but reduced the ability to recognize sadness, regardless of the type of stimulus (static vs. moving). Convergent with the idea of hierarchical involvement of the vagus in establishing safety, as put forward by the Polyvagal theory, we argue that our findings may be explained by vagus-evoked differential adjustment strategies to emotional expressions. Taken together, our findings fit with an evolutionary perspective on the vagus nerve and its involvement in emotion recognition for the benefit of survival.

## Introduction

Successful social interactions, beneficial for survival (Fischer & Manstead, 2008), rely on our ability to recognize and respond to other’s emotions (Frijda & Mesquita, 2004; Frith, 2009). The study of emotion recognition has traditionally focused on emotions as derived from faces (Feldman Barrett et al., 2011). Although humans tend to report relying on facial expressions of emotion in judging emotional expressions, the information that people deduct from facial expressions has been observed to rely on bodily expressions, also known as illusory facial affect (Aviezer et al., 2012; De Gelder, 2006; Kret & de Gelder, 2013; Meeren et al., 2005; Rajhans et al., 2016; Van den Stock et al., 2007). This may be explained by the idea that body expressions are the most evolutionarily preserved, providing an instant means to communicate emotional information (De Gelder, 2006).

Regarding an evolutionary perspective on emotion recognition and social engagement with our environment, it has been argued that the vagus nerve is the driving phylogenetic element (Porges, 2001, 2003, 2007, 2009). According to Porges’ Polyvagal theory (Porges, 2001, 2003, 2007), mammals—as opposed to reptiles and fish—develop a ventral, myelinated, branch of the vagus, whose activity has been specifically linked to the ability to monitor and regulate complex behaviors, such as attention, motion, emotion, and communication (Porges, 2001, 2003, 2007, 2009). Further elaboration on how the vagus may be involved in such complex behaviors has been provided by the neurovisceral integration model (Thayer & Lane, 2000, 2009), putting forward that (cardiac) vagal tone may reflect functional integrity of the neural networks underlying emotion-cognition interactions (i.e., predominantly the central autonomic network (CAN) comprising the anterior cingulate-, insular-, and ventromedial prefrontal cortices, (parts of) the amygdala and hypothalamus, and the nucleus of the solitary tract). Importantly, according to their model, the efferent influences of, and afferent peripheral signals received by, those neural networks are mediated by the vagus nerve (Thayer & Lane, 2000). Indeed, vagal activity has been found to be related to empathy, recognizing emotions, and prosocial behavior (Beauchaine, 2001; Butler et al., 2006; Eisenberg et al., 1997; Kogan et al., 2014; Kok & Fredrickson, 2010; Oveis et al., 2009; Porges, 2001; Quintana et al., 2012; Wang et al., 2013).

Further causal evidence for such a role comes from studies applying transcutaneous stimulation to the vagus nerve (tVNS), which has been reported to enhance the ability to infer emotions from the eye region (Colzato et al., 2017) and faces, but not static bodies in healthy individuals (Koenig et al., 2019; Sellaro et al., 2018). It has been argued that the apparent dissociation between facial versus bodily expressions might be explained by considering that the vagus directly influences cranial nerves regulating gaze orienting and facial expressions as, for example, allowing eye contact or enhancing gaze detection (Maraver et al., 2020).

Another consideration, however, is that static expressions have little ecological validity and are processed differently from dynamic expressions (Atkinson et al., 2004; Braddick, 1992; Oram & Perrett, 1994). That is, static expressions correspond to a peak in movement, therefore providing sufficient information to identify at least the basic emotions (Atkinson et al., 2004; Calvo & Lundqvist, 2008; de Gelder & Van den Stock, 2011; Ekman, 1972; Ekman & Friesen, 1978; Lundqvist et al., 1998; Sprengelmeyer et al., 1999). However, dynamic properties (i.e., time course) of emotional expressions are known to influence the perceived intensity of an emotion and the ability to recognize these as such (Pollick et al., 2003). In other words, inferring an emotional state at least partly relies on the meaning as generated by movement (Atkinson et al., 2004). Perhaps even more for bodies, as consensus holds that a combination of static and dynamic information is most effective in distinguishing emotional expressions in bodies when no other cues (such as face, voice, or context) are available (Atkinson et al., 2004).

Therefore, the purpose of the current study was to investigate whether the stimulation of the afferent vagus (by means of tVNS) enhances the recognition of emotions from bodies, taking into account the role of dynamic information. Our first goal was to replicate the (null) finding observed in static bodies from the study by Sellaro et al. (2018) and, additionally, to test whether their results generalize to situations in which static and dynamic, or only dynamic, information can be inferred. The recognition of static bodies was found not to be affected by tVNS (Sellaro et al., 2018). It seems plausible to assume that the afferent ventral vagus, stimulated by tVNS, might regulate the detection of emotion from moving, but not/more than from static bodies for two main reasons. First, the polyvagal theory (Porges, 1995, Porges, 2001, 2003, 2007, 2009) proposes that the ventral and dorsal branches of the vagus nerve are hierarchically (i.e., ventral activation suppresses dorsal activation and dorsal activation equals ventral inactivation, Porges, 2001; 2009) and distinctly involved in influencing behavior and specifically in monitoring (the need for) movement, mobilization, and (social) engagement with the environment. That is, activation of the dorsal vagal complex is suggested to be involved in immobilizing and (socially) withdrawing behaviors. Studies involving vagus nerve interventions (e.g., stimulation, vagotomy) commonly fail to distinguish between ventral and dorsal branches and/or report the intervention site. However, animal studies have shown, for example, that temporary *in*activation of the dorsal vagal complex by means of a reversible lesion decreased anxiety-like nonengagement with the environment (e.g., assessed by the distance of area explored and number of visits to the center of the open field) behavior in the open-field test (Miller et al., 2002) and prevents depressive-like, immobilizing, effects of inflammation (Marvel et al., 2004). Directly activating ventral afferent fibers resulted in similar effects as inactivating the dorsal vagus; surgical ventral vagal nerve stimulation resulted in enhanced extinction of conditioned fear by means of less freezing (i.e., more movement and engagement with the environment) (Peña et al., 2013). Surgical ventral VNS antianxiety and/or antidepressive effects also have been reported in epilepsy patients (Elger et al., 2000) and depressed patients with mild, but not extreme, antidepressant resistance (Sackeim et al., 2001, for a review see Carreno & Frazer, 2017). Together, these studies seem to support ventral vs. dorsal involvement as put forward by Porges (1995, 2001, 2003, 2007, 2009); the ventral vagal branch may be mainly involved in adaptive mobilization and (social) engagement with the environment. Because adaptiveness to a dynamic environment is presumably more complex and requires more monitoring than in case of a static environment, the involvement of the ventral vagus may be more pronounced when exposed to dynamic stimuli. A second reason one might expect an affect regarding moving, but not static, bodies is that the combination of static and dynamic information has been proposed to be more informative when deriving emotional information from bodies and creates a more ecologically valid situation (Aronoff et al., 1992; de Meijer, 1989; Dittrich et al., 1996).

To test the possible differential influence of tVNS on static versus moving stimuli, full-light displays (FLDs) of a body were used to present a combination of static and dynamic information; the static picture of a full-light displayed body can still be perceived as a body, thus providing meaningful information (Atkinson et al., 2004). To distinguish between information derived from static form and information derived from movement, Johansson (1973) developed point-light displays (PLDs), in which the movement of a figure (i.e., a body) is represented by a number of illuminated patches that highlight the movement of main body parts. When static, these patches only represent a seemingly meaningless configuration. When moving, these patches can be transformed into a configuration of a moving body (Johansson, 1973). Indeed, PLDs have been found to be sufficient to identify basic emotions expressed by the body movements (Brownlow et al., 1997; Dittrich et al., 1996; Humphreys et al., 1993; Pollick et al., 2003). However, the ability to recognize emotions from such displays differs between the corresponding emotional valence (Actis-Grosso et al., 2015; Atkinson et al., 2004), possibly stressing the need to take into account the nature of the presented emotion instead of evaluating the general emotion recognition process. This is furthermore supported by Porges (2001), explaining that relations of physiology to emotions may depend on the specific emotion studied. Indeed, as pointed out by Rainville et al. (2006), basic emotions are associated with distinct patterns of cardiorespiratory activity linked to the vagus nerve activity (Rainville et al., 2006). However, previous finding with tVNS have shown no specific enhancing effect as a function of the type of emotion in healthy individuals (Colzato et al., 2017; Koenig et al., 2019; Sellaro et al., 2018), and therefore, we do not have a specific hypothesis regarding the direction in which particular emotions might or might not show an effect.

In summary, we expected tVNS to enhance the ability to detect emotions expressed by dynamic (i.e., FLD and PLD), but not static displays of bodies. If the processing of static and dynamic information is affected by the vagus only when static and dynamic information can be inferred, an effect of tVNS should only be visible for FLDs, but not PLDs. Moreover, Sellaro et al. (2018) put forward the idea that the effect of tVNS might be influenced by baseline vagal tone, as indexed by vagally mediated resting-state heart rate variability (HRV). Taken both findings together and to test this possibility, we assessed relevant vagally mediated HRV indices (Laborde et al., 2017): namely, the root mean square of the successive differences of interbeat intervals (RMSSD, i.e. a well-validated measure of HRV; Berntson et al., 1997; Malik, 1996); the number of pairs of successive interbeat intervals that differ more than 50 ms (NN50); and absolute power of the high-frequency band (HF, 0.15-0.1 Hz). However, while HRV represents a measure of efferent vagal tone, tVNS stimulates the afferent vagal pathway. Therefore, exploring the role of HRV is a secondary hypothesis and our focus is placed on the effect of tVNS in recognizing different emotions displayed by static or moving bodies.

## Materials and Methods

### Participants

The required sample size was estimated based on previous protocols and observed effect sizes (Colzato & Steenbergen, 2017; Sellaro et al., 2018), the number of factors, levels thereof, and covariates in the current design, while allowing modest attrition. Seventy-five healthy individuals gave consent for participation. Two participants did not meet the health criteria for participation and were therefore not further included. Therefore, 73 healthy individuals (58 females, 15 males, mean age = 20.53, SD = 2.03, range = 18-28) participated in the current experiment. Participants were recruited through an online recruitment system, calling for volunteers to participate in a two-session study on the effect of brain stimulation on social decision-making in exchange for course credit or a small monetary reward. Individuals were screened using criteria based on the Mini International Neuropsychiatric Interview (M.I.N.I.; Sheehan et al., 1998) to confirm eligibility with regard to the absence of a variety of disorders and drug use (Colzato et al., 2005; Colzato et al., 2008). Following previous protocols (Jongkees et al., 2018; Sellaro et al., 2018; Steenbergen et al., 2020), participants were considered eligible if they met the following criteria: age between 16 and 30 years old; no self-reported excessive (>25 per week) alcohol use; no use of soft or hard drugs in the past month; no pregnancy; no gastrointestinal disease; no cardiovascular disease; no use of any psychoactive medication; no mental or physical disability that will hinder participation; no history of neurological or psychiatric disorders; no history of brain surgery; no intracranial metal implants; no pacemaker or other implanted device; no history of stroke; not recent experience of or susceptibility to fainting or panic attacks; no brain injury; no claustrophobia; no epilepsy or first-degree relative with epilepsy; no susceptibility to dizziness or headaches; and no skin abnormality, such as eczema, in the left ear.

All participants received verbal and written explanation of the procedure and possible side effects (i.e., tingling or itching sensation of the skin below the electrodes, muscle contractions, headache, or skin below the electrodes turning red) and signed, informed consent before starting the procedure. To minimize expectation effects, no information was provided about the type of stimulation applied in each specific session or the expected direction of effects. The procedures conformed to the ethical standards of the 1975 Declaration of Helsinki (World Health Organisation, 2013), and subsequent amendments, and were approved by the local ethics committee (CEP17-1220427, Psychology Research Ethics Committee, Institute for Psychological Research, Leiden University).

1.1. **Procedure**

**Fig. 1:**
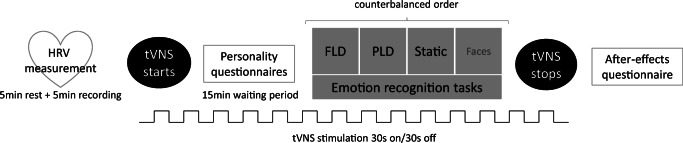
Flowchart of the structure of the experimental sessions. tVNS stimulation condition (active vs. sham) was counterbalanced across participants.

Sham and active tVNS were applied in two counterbalanced, sessions separated by at least 7 days. Upon arrival to the first session, participants read and signed the informed consent, after which their length and weight (i.e., using an OMRON scale) were assessed such that body mass index (BMI) could be calculated. Participants were then individually placed in a sound-attenuated cubicle and instructed to turn off all mobile and Bluetooth devices they carried. Next, participants were asked to apply a Polar H7 chest belt to record interbeat intervals. After a 5-minute resting period, a Samsung Galaxy Tab 10 tablet was used to run the Elite HRV app to wirelessly record heart-rate data for 5 minutes. During these 10 minutes, participants were instructed to sit still and breathe spontaneously; although controlling for the influence of respiration rate on HRV remains a topic of debate (Laborde et al., 2017), respiration rate does not modulate HRV in resting-state measurements in healthy individuals (Denver et al., 2007; Quintana et al., 2016). After completion of these measurements, following previous protocols (Jongkees et al., 2018; Sellaro et al., 2018; Steenbergen et al., 2020), stimulation was applied from 15 minutes before the start of the tasks until their completion. Consistent with Sellaro et al. (2018), during the 15-minute waiting period, participants filled out a number of personality questionnaires to infer mood (positive and negative affect scale, PANAS; Watson et al., 1988), interpersonal reactivity (Interpersonal Reactivity Index, IRI; Davis, 1980, 1983), empathy (the Empathy Quotient, EQ; Baron-Cohen & Wheelwright, 2004), autistic traits (Autistic Quotient, AQ; Baron-Cohen et al., 2001), and alexithymia (Bermond-Vorst Alexithymia Questionnaire, Vorst & Bermond, 2001). Whereas the PANAS was filled out in both sessions, the other questionnaires are assumed to reflect trait measures and were filled out only once. Participants were asked to perform four tasks, three of which will be evaluated in light of the current study. Performance on a fourth task (a facial emotion recognition task) will be reported elsewhere. The order of the four tasks was counterbalanced but kept constant over the two sessions. After completion of these tasks, participants filled out a questionnaire rating, on a scale from 1 to 5, a number of possible side-/aftereffects. That is, headache, neck pain, nausea, stinging sensation under the electrodes, burning sensation under the electrodes, muscle contractions in the neck or face, and generic uncomfortable feelings. Upon completion of the second session, participants were debriefed and reimbursed. Figure 1 depicts a flowchart of the structure of the experimental sessions.

1.2. **Questionnaires**

The following battery of personality questionnaires has been previously used in similar tVNS procedures (Sellaro et al., 2018), and our rationale to include them is to control for nonspecific effects of tVNS on levels of empathy at baseline or possible autistic traits that could emerge in our sample of healthy participants. To measure the reactions of participants to the observed experiences of others, we used the Interpersonal Reactivity Index (Davis, 1980, 1983); a 28-item questionnaire in which items are rated on a 5-point Likert scale ranging from 0 (“Does not describe me well”) to 4 (“Describes me very well”). The questionnaire consists of four subscales, each comprising seven different items. The tendency to spontaneously adopt the psychological point of view of others is captured in Perspective Taking, whereas the Fantasy subscale assessed participants’ tendencies to transpose themselves imaginatively into the feelings and actions of fictive characters in books, movies, and plays. Empathic Concern captures other-oriented feelings of sympathy and concerns for unfortunate others, whereas Personal Distress captures self-oriented feelings of personal anxiety and unease in tense interpersonal settings. The total score ranges from 0 to 112, and subscale scores range from 0 to 28, each with higher scores indicating more interpersonal reactivity (Davis, 1980; Pulos et al., 2004).

The 60-item Empathy Quotient (EQ; Baron-Cohen & Wheelwright, 2004) was used to assess the ability to understand what others are feeling and why, and to what degree participants are affected hereby (i.e., empathy). Participants answer to what extent they agree with each of the 60 statements, choosing between “strongly agree,” “slightly agree,” “slightly disagree,” and “strongly disagree.” Scores range between 0 and 80, with higher scores indicating more empathy.

To assess autistic-like traits, participants filled out the 50-item Autism Quotient (AQ; Baron-Cohen et al., 2001). Participants rate, for each statement, to what extent they agree. A single score, ranging between 0 and 50 is derived, for which higher scores indicate more autistic-like traits.

The BVAQ-40 (Vorst & Bermond, 2001) is a 40-item questionnaire to assess difficulties in one’s ability to identify own emotions (i.e., alexithymia). Participants rate, on a scale from 1 (completely) to 5 (not at all), to what extent a certain statement applied to them. The items add up to two scores allowing a distinction between cognitive versus affective understanding of one’s emotions, and 5 subscores: Emotionalizing, Fantasizing, Identifying, Analyzing, and Verbalizing. Importantly, higher scores indicate a more difficulty in identifying one’s own emotions.

In order to assess mood state, we used the positive and negative affect scale (PANAS; Watson et al., 1988). The PANAS includes two 10-item mood scales (i.e., 20 in total) briefly assessing positive and negative affect. Participants are asked to rate the extent to which they experience each of the 20 presented emotions at this moment, using a 5-point scale. A positive and a negative mood score is obtained by adding the respective items, both ranging from 10 to 50.

1.3. **Transcutaneous Vagus Nerve Stimulation (tVNS)**

Following previous protocols (Beste et al., 2016; Colzato et al., 2017; Jongkees et al., 2018; Sellaro et al., 2018; Steenbergen et al., 2015), the NEMOS^®^ tVNS instrument, consisting of two titan electrodes attached to a gel frame and connected to a wired neurostimulating device (CM02, Cerbomed, Erlangen, Germany) was used to stimulate vagal afferents, located at the cymba concha, of the left ear. The device was programmed to a stimulus intensity at 0.5 mA with a stimulation frequency of 25 Hz delivered in pulses of 200-300 μs. Stimulation was active for 30 sec, followed by a break of 30 sec. Following Kraus et al. (2007), in the sham condition, the stimulation electrodes were attached to the center of the left ear lobe instead of the left cymba concha for stimulation. Since efferent fibers of the vagus nerve modulate cardiac function, cardiac safety has always been a concern in the therapeutic use of vagus nerve stimulation (Kraus et al., 2007; Sperling et al., 2010). Efferent vagal fibers to the heart are supposed to be located on the right side (Nemeroff et al., 2006). In order to avoid cardiac side effects, electrode placement is therefore always performed on the left side (Kreuzer et al., 2012; Nemeroff et al., 2006).

1.4. **Heart Rate Variability**

A Polar H7 heart rate monitoring system (Polar Electro, Kempele, Finland), which wirelessly receives heart rate (HR) data from a chest belt applied and worn by the participants, was used to assess resting-state interbeat intervals (IBI) for 5 minutes (see also Colzato, Jongkees, et al., 2018; Colzato & Steenbergen, 2017). Polar H7 has been validated for the recording of IBI (Weippert et al., 2010). To extract raw data, the Elite HRV application (https://elitehrv.com) was used. Text files retrieved from Elite HRV were subsequently imported into Kubios (premium version 3.0, 2017, Biosignal Analysis and Medical Imaging Group, University of Kuopio, Finland; Tarvainen et al., 2014). To filter out artifacts, we used the automatic thresholding procedure. Subsequently, average heart rate (HR) in beats per minute (BPM) was retrieved, as well as relevant vagally mediated HRV indices (Laborde et al., 2017); RMSSD, calculated as the root mean square of successive differences of inter beat intervals, the number of pairs of successive inter beat intervals that differ more than 50 ms (NN50), and absolute power of the high-frequency band (HF, 0.15-0.1 Hz).

1.5. **Body emotion recognition tasks**

The ability to recognize emotions expressed by bodies was assessed by means of three tasks differing with regard to the type of stimuli that had to be evaluated: static bodies, full-light body displays (FLDs), and point-light body displays (PLDs). The order of the tasks was counterbalanced but kept constant over the two sessions. E-prime 2.0 software (Psycholoy Software Tools, Inc., Pittsburgh, PA) was used to program the task, present the stimuli, and collect participants’ responses. For all tasks, participants were presented with randomly ordered stimuli in the middle of the computer screen and asked to use the mouse to indicate which of the four presented emotions (i.e., anger, fear, happiness, sadness) best described the emotion the body was depicting. These four emotions were displayed at the corners of an imaginary square around the stimulus. Stimuli remained on the screen until a participant responded, no response deadline was applied. Trials were separated by a blank screen presented for 500 ms. The static body emotion recognition task was adapted from Sellaro et al. (2018); 80 (20 for each emotion) black-and-white stimuli were selected from the Bodily Expression Action Stimulus Test (BEAST; de Gelder & Van den Stock, 2011).

For the FLD and PLD tasks, stimuli were taken from Atkinson et al. (2004). Both tasks included 40 trials (10 unique videos per emotion; anger, fear, happiness, and sadness). Previous studies using a similar procedure with the same number of videos (Actis-Grosso et al., 2015) or even less (Siqi-Liu et al., 2018) have shown reliable results. For both tasks, stimuli consisted of 3-second movie clips derived from the same recording. After 3 seconds, the recording remained static until participants responded. In each session, participants were hence presented 160 trials: 80 static trials (20 per emotion) and 80 moving trials [2 tasks (PLD vs. FLD) × 4 emotions (happiness vs. sadness vs. angry, vs. fear) × 10 stimuli (unique videos)]. The exact same tasks were repeated in the second session.

1.6. **Statistical analyses**

Analyses were performed using SPSS 23.0 for Windows. The dependent variable for all three tasks was participants’ accuracy in recognizing emotional expressions. Given that emotion recognition tasks were originally developed to index impairments in emotion recognition skills in adults suffering from autism spectrum disorders, and in line with previous studies (Colzato et al., 2017; Domes et al., 2007; Sellaro et al., 2018), we bypassed eventual ceiling effects in healthy subjects by dividing the items into two subsets of easy and difficult items. Following Sellaro et al. (2018), stimuli were labelled “easy” or “difficult” based on the median-split of item difficulty as derived from the data provided by the BEAST (de Gelder & van den Stock, 2011) for the static bodies and by Atkinson’s dataset for the PLDs and FLDs (Atkinson et al., 2004; 2012). A 3×4×2×2 repeated measures ANOVA (rmANOVA) was performed, all with type of stimuli (FLD vs. PLD vs. Static), emotion (anger vs. fear vs. happiness vs. sadness), difficulty (easy vs. difficult), and session (sham vs. active) as within-subject factors. Analyses were repeated adding RMSSD, NN50, and HF as covariates, as suggested by Sellaro et al. (2018) and recommended by (Laborde et al., 2017). In case of violation of the sphericity assumption, Greenhouse-Geisser correction was applied, and corrected values are reported. A significance level of *p* < 0.05 was adopted for all statistical tests. In case of significant effects, post-hoc pairwise comparisons were performed, and we report Bonferroni-corrected *p*-values (i.e., *p*-values multiplied by the number of tests performed) to interpret the direction of effects.

## Results

One participant dropped out after the first session and therefore was excluded from further analyses. Due to unknown circumstances, possibly technical issues and/or issues in applying the chest belt, HR recordings were missing or fell out of the range normally observed over lifetime (i.e., 7 ≤ RMSSD ≥ 103; Umetani et al., 1998) for four participants in the active session, for a different six participants in the sham session, and for two participants in both sessions. Because of the relationship between HRV and emotion regulation (Mather & Thayer, 2018), we considered our sample as whole by including those who had valid HRV and behavioral data in the emotion recognitions tasks. Therefore, we performed the analyses in the resulting sample of 60 valid participants (49 females, 11 males, *M*_age_ = 20.38, *SEM*_age_ = 0.24, *M*_BMI_ = 23.34, *SEM*_BMI_ = 0.38).

1.7. **Personality questionnaires**

Participants scores on the questionnaires tapping into interpersonal reactivity, trait empathy, autistic traits, and alexithymia fell within the normal range and, where applicable, are comparable to those observed by Sellaro et al. (2018): IRI_total_ (*M* = 67.40, *SEM* = 1.65), IRI_PerspectiveTaking_ (*M* = 18.67, *SEM* = 0.54), IRI_FantasyScale_ (*M* = 17.53, *SEM* = 0.69), IRI_EmpathicConcern_ (*M* = 18.78, *SEM* = 0.46), IRI_PersonalDistress_ (*M* = 12.42, *SEM* = 0.59), EQ_total_ (*M* = 47.48, *SEM* = 1.39), AQ_total_ (*M* = 13.15, *SEM* = 0.87), BVAQ_AffectiveDimension_ (*M* = 38.18, *SEM* = 1.18), BVAQ_CognitiveDimension_ (*M* = 53.48, *SEM* = 1.70).

1.8. **Mood and After tVNS effects**

No differences in mood or after effects as a function of session (sham vs. active) were observed, all *p*_s_ ≥ 0.07. However, participants were less accurate in reporting which stimulation type they thought to have received in the sham as compared to the active session, *t*(59) = −3.39, *p* < 0.01, , M_diff_ = −0.23, 95% confidence interval (CI) [−0.09, 0.37] (Table 1).

**Table 1:** Mean ± standard error of the mean of self-reported positive and negative affect and after-effects as observed in both sessions.

	Sham	Active
Positive affect	36.35 ± 0.64	36.35 ± 0.66
Negative affect	20.68 ± 0.76	20.33 ± 0.76
Headache	1.37 ± 0.09	1.32 ± 0.07
Neck pain	1.15 ± 0.07	1.20 ± 0.06
Nausea	1.10 ± 0.04	1.17 ± 0.06
Muscle contractions	1.18 ± 0.07	1.30 ± 0.09
Stinging sensation	3.35 ± 0.17	3.42 ± 0.17
Burning sensation	2.27 ± 0.17	2.27 ± 0.17
Generic uncomfortable feeling	2.18 ± 0.16	2.38 ± 0.15
Accuracy reporting stimulation type*	0.27 ± 0.06	0.50 ± 0.06

After effects were rated on a scale ranging from 1 (not at all) to 5 (very much). *p < 0.01

3.3. **HRV**

As resting-state heart rate variability was measured at the beginning of both sessions, before starting stimulation, we took the average to come to a more reliable baseline measurement. Paired samples t-tests demonstrated no significant differences in HRV measures, neither for time-domain measures nor for frequency domain measures, all *p*_s_ ≥ 0.78 (Table 2).

**Table 2:** Mean ± standard error of the mean of the heart rate variability measures in the domain of time and frequency

	Sham	Active
Average HR in BPM	82.73 ± 1.38	82.37 ± 1.44
RMSSD	36.72 ± 2.62	36.52 ± 2.53
NN50	55.50 ± 7.34	54.28 ± 6.98
HF power in ms^2^	727.92 ± 102.18	706.29 ± 88.61

3.4. **Emotion recognition tasks**

rmANOVA performed on accuracy in identifying emotions as a function of the type of stimuli (FLD vs. PLD vs. static), emotion (anger, fear, happiness, sadness), item difficulty (easy vs. difficult), and tVNS session (sham vs. active) revealed different significant sources of variance (see Table 3 for the rmANOVA effects and Table 4 for the Bonferroni-corrected post hoc comparisons).

**Table 3:** Inferential statistics for the repeated-measures ANOVA on accuracy as a function of type of stimuli (FLD vs. PLD vs. static), emotion (anger vs. fear vs. happiness vs. sadness), item difficulty (easy vs. difficult) and stimulation condition (active vs. sham tVNS)

rmANOVA effect	F	*p*	η_p_^2^	MSE
**Stimulus type**	**13.26**	**<0.01**	**0.18**	**0.53**
**Emotion**	**22.42**	**<0.01**	**0.27**	**2.43**
**Difficulty**	**44.13**	**<0.01**	**0.43**	**1.00**
tVNS session	0.11	0.74	<0.01	<0.01
**Stimulus type × Emotion**	**53.41**	**<0.01**	**0.47**	**5.47**
**Anger: FLD vs. PLD vs. Static**	**43.48**	**<0.01**	**0.42**	**0.23**
**Fear: FLD vs. PLD vs. Static**	**9.04**	**<0.01**	**0.13**	**0.03**
**Happiness: FLD vs. PLD vs. Static**	**53.69**	**<0.01**	**0.48**	**2.26**
**Sadness: FLD vs. PLD vs. Static**	**12.29**	**<0.01**	**0.17**	**0.05**
**Stimulus type × Difficulty**	**24.88**	**<0.01**	**0.30**	**0.54**
**Easy: FLD vs. PLD vs. Static**	**19.52**	**<0.01**	**0.25**	**0.04**
**Difficult: FLD vs. PLD vs. Static**	**17.24**	**<0.01**	**0.22**	**0.10**
**Emotion × Difficulty**	**7.32**	**<0.01**	**0.11**	**0.21**
Stimulus type × tVNS session	0.29	0.74	<0.01	<0.01
Emotion × tVNS session*	**4.54**	**<0.01**	**0.07**	**0.10**
Difficulty × tVNS session	0.38	0.54	<0.01	<0.01
**Stimulus type × Emotion × Difficulty**	**9.60**	**<0.01**	**0.14**	**0.31**
**FLD: Emotion × Difficulty**	**10.27**	**<0.01**	**0.15**	**0.10**
PLD: Emotion × Difficulty	1.07	0.36	0.02	0.02
**Static: Emotion × Difficulty**	**16.60**	**<0.01**	**0.22**	**0.42**
Stimulus type × Emotion × tVNS session	1.11	0.35	0.02	0.03
Stimulus type × Difficulty × tVNS session	0.25	0.78	<0.01	<0.01
Emotion × Difficulty × tVNS session	1.36	0.26	0.02	0.02
Stimulus type × Emotion × Difficulty × tVNS session	0.47	0.77	<0.01	<0.01

Significant effects are highlighted in bold font and asterisk highlights the main interaction effect between emotion and tVNS stimulation.

**Table 4:** Inferential statistics for the pairwise Bonferroni-adjusted post hoc comparisons of the significant effect

Pairwise comparisons	M_1_ ± SEM_1_	M_2_ ± SEM_2_	Mdiff	*p*	95% CI
*Stimulus type*					
**FLD - PLD**	**0.93 ± 0.01**	**0.90 ± 0.01**	**0.03**	**<0.01**	**0.02, 0.05**
**FLD - Static**	**0.93 ± 0.01**	**0.89 ± 0.01**	**0.04**	**<0.01**	**0.02, 0.06**
PLD - Static	0.90 ± 0.01	0.89 ± 0.01	<0.01	1.00	−0.02, 0.03
*Emotion*					
**Fear - Anger**	**0.94 ± 0.01**	**0.91 ± 0.01**	**0.03**	**0.01**	**0.00, 0.05**
**Fear - Happiness**	**0.94 ± 0.01**	**0.84 ± 0.01**	**0.10**	**<0.01**	**0.05, 0.14**
Fear - Sadness	0.94 ± 0.01	0.93 ± 0.01	0.01	1.00	−0.01, 0.03
**Anger - Happiness**	**0.91 ± 0.01**	**0.84 ± 0.01**	**0.07**	**<0.01**	**0.02, 0.12**
Anger - Sadness	0.91 ± 0.01	0.93 ± 0.01	0.01	1.00	−0.01, 0.03
**Happiness - Sadness**	**0.84 ± 0.01**	**0.93 ± 0.01**	**−0.09**	**<0.01**	**−0.13, −0.04**
*Difficulty*					
**Easy - Difficult**	**0.92 ± 0.01**	**0.89 ± 0.01**	**0.04**	**<0.01**	**0.02, 0.05**
*Stimulus type × Emotion*					
**Anger: FLD - PLD**	**0.90 ± 0.01**	**0.86 ± 0.01**	**0.04**	**0.02**	**0.00, 0.07**
**Anger: FLD - Static**	**0.90 ± 0.01**	**0.98 ± 0.01**	**0.08**	**<0.01**	**0.05, 0.11**
**Anger: PLD - Static**	**0.86 ± 0.01**	**0.98 ± 0.01**	**0.12**	**<0.01**	**0.09, 0.16**
**Fear: FLD - PLD**	**0.95 ± 0.01**	**0.91 ± 0.01**	**0.04**	**<0.01**	**0.01, 0.06**
Fear: FLD - Static	0.95 ± 0.01	0.95 ± 0.01	<0.01	1.00	0.01, 0.06
**Fear: PLD - Static**	**0.91 ± 0.01**	**0.95 ± 0.01**	**0.03**	**<0.01**	**−0.05, −0.01**
**Happiness: FLD - PLD**	**0.95 ± 0.01**	**0.90 ± 0.01**	**0.05**	**<0.01**	**0.01, 0.08**
**Happiness: FLD - Static**	**0.95 ± 0.01**	**0.67 ± 0.03**	**0.28**	**<0.01**	**0.20, 0.36**
Sadness: FLD - PLD	0.92 ± 0.01	0.90 ± 0.01	0.01	0.63	−0.01, 0.04
**Sadness: FLD - Static**	**0.92 ± 0.01**	**0.96 ± 0.01**	**0.04**	**<0.01**	**0.01, 0.07**
**Sadness: PLD - Static**	**0.91 ± 0.01**	**0.96 ± 0.01**	**0.06**	**<0.01**	**0.02, 0.09**
*Stimulus type × Difficulty*					
Easy: FLD - PLD	0.93 ± 0.01	0.94 ± 0.01	0.01	0.36	−0.02, 0.00
**Easy: FLD - Static**	**0.93 ± 0.01**	**0.90 ± 0.01**	**0.03**	**<0.01**	**−0.06, −0.01**
**Easy: PLD - Static**	**0.94 ± 0.01**	**0.90 ± 0.01**	**0.04**	**<0.01**	**−0.06, −0.02**
**Difficult: FLD - PLD**	**0.93 ± 0.01**	**0.85 ± 0.01**	**0.08**	**<0.01**	**0.05, 0.10**
**Difficult: FLD - Static**	**0.93 ± 0.01**	**0.83 ± 0.01**	**0.04**	**<0.01**	**0.01, 0.08**
Difficult: PLD - Static	0.85 ± 0.01	0.83 ± 0.01	0.03	0.07	−0.07, 0.00
*Emotion × Difficulty*					
Anger: easy - difficult	0.91 ± 0.01	0.92 ± 0.01	0.01	0.34	−0.04, 0.01
**Fear: easy - difficult**	**0.96 ± 0.01**	**0.92 ± 0.01**	**0.04**	**<0.01**	**−0.06, −0.02**
**Happiness: easy - difficult**	**0.88 ± 0.01**	**0.80 ± 0.02**	**0.08**	**<0.01**	**−0.11, −0.05**
Sadness: easy - difficult	0.93 ± 0.01	0.92 ± 0.01	0.01	0.20	−0.03, 0.00
*Stimulus type × Emotion × Difficulty*					
FLD - Anger: easy - difficult	0.89 ± 0.01	0.91 ± 0.01	0.02	0.26	−0.02, 0.06
**FLD - Fear: easy - difficult**	**0.99 ± 0.01**	**0.91 ± 0.01**	**0.08**	**<0.01**	**0.11, 0.05**
FLD - Happiness: easy - difficult	0.95 ± 0.01	0.94 ± 0.02	<0.01	0.64	−0.05, 0.03
**FLD - Sadness: easy - difficult**	**0.90 ± 0.02**	**0.94 ± 0.01**	**−0.04**	**<0.01**	**0.02, 0.07**
**Static - Anger: easy - difficult**	**0.97 ± 0.03**	**0.99 ± 0.01**	**0.02**	**<0.01**	**0.01, 0.03**
**Static - Fear: easy - difficult**	**0.92 ± 0.01**	**0.98 ± 0.01**	**0.06**	**<0.01**	**0.04, 0.08**
**Static - Happiness: easy - difficult**	**0.73 ± 0.03**	**0.60 ± 0.04**	**0.13**	**<0.01**	**−0.20, −0.06**
Static - Sadness: easy - difficult	0.96 ± 0.01	0.95 ± 0.01	0.01	1.11	−0.03, 0.01

Significant comparisons are highlighted in bold font.

First, the rmANOVA revealed a main effect of type of stimuli, for which Bonferroni-adjusted post-hoc tests indicated that accuracy for the FLD items was significantly higher than for the PLD and static items. The accuracy between PLD and Static items did not differ. Second, we found a main effect of emotion for which pairwise comparisons revealed that accuracy for fearful bodies was higher than for angry and happy, but not from sad items. Accuracy for angry bodies also was higher than happy, but not different from sad bodies, whereas happy were recognized worse than sad items. Third, we found a significant main effect of item difficulty, for which Bonferroni-adjusted pairwise comparisons showed that easy items led to better performance than difficult items. Finally, the main effect of tVNS session did not reach significance.

Several two-way interactions turned out significant. First, we observed an interaction between type of stimuli and emotion. Within emotion pairwise comparisons revealed that for angry items, accuracy was higher for the static bodies compared to the FLD and PLD. Similarly, accuracy for FLD was higher than for PLD. For fearful items, FLD bodies were recognized better than PLD but similar to static bodies, and accuracy between PLD and static bodies also differed. Happy FLD bodies were recognized better than PLD and static bodies, as well as PLD performance was better than for the static items. Finally, for sad items, accuracy for static bodies was higher than for FLD and PLD, while performance between FLD and PLD did not differ. Second, the type of stimuli and item difficulty interaction proved to be significant. Within level of difficulty pairwise analysis, showed that for the easy items, accuracy for FLD and PLD was similar, but lower for the static bodies compared with FLD and PLD. For difficult items, FLD bodies were recognized better than PLD and static items, but PLD and static bodies did not differ. Third, the interaction between emotion and item difficulty followed by within emotion pairwise comparisons showed that, while no difference between easy and difficult items was observed for angry and sad items, for fearful and happy bodies accuracy was higher for easy than for difficult items.

Furthermore, we observed a three-way interaction between type of stimuli, emotion, and item difficulty. Post-hoc within stimulus type analysis derived a significant interaction between emotion and item difficulty for the FLD items. Bonferroni-corrected pairwise comparisons showed that fearful easy items were recognized better than difficult ones, while the opposite was observed for sad bodies, and no differences were observed for angry and happy items. No significant interaction between emotion and item difficulty was observed for PLD items. However, for the static items, we also found a significant emotion by item difficulty interaction. Bonferroni-adjusted pairwise comparisons showed that for angry and fearful items, difficult items were recognized better than easy items. For happy items, the opposite pattern was observed, and no significant differences were found for sad static bodies.

Finally, regarding our main hypothesis about the effects of tVNS in emotion recognition, we observed a significant two-way interaction between emotion and session. Pairwise Bonferroni-corrected within emotion analysis showed that, for angry items, accuracy for active tVNS was higher than for the sham *t*(59) = 2.85, *p* < 0.01, M_diff_ = 0.03, 95% CI [0.01, 0.05]), whereas for sad bodies, the pattern was the opposite *t*(59) = 2.24, *p* = 0.03, M_diff_ = −0.02, 95% CI [−0.03, 0.00]), and no differences between active and sham tVNS were observed for fearful and happy items (*p*s > 0.28; Table 5). Because of the sphericity violation in our results, we also ran multivariate test statistics since they are not dependent on the sphericity assumption (O’Brien & Kaiser, 1985). Although there is a trade-off in test power between univariate and multivariate approaches (Stevens, 2002), MANOVA results confirmed the interaction effect between tVNS session and emotion recognition (*F*(3,57) = 6.30, *p* < 0.004; Wilks' Λ = 0.75).

**Table 5:** Proportion of correct answers (accuracy) on the body emotion recognition tasks for the active and sham sessions for the three emotional tasks (FLD, PLD, and static bodies) the average of them, and the four presented emotions (anger, fear, happiness, sadness)

	Sham tVNS				Active tVNS			
	FLD	PLD	Static	Average	FLD	PLD	Static	Average
Anger	0.92 ± 0.01 (0.66-1)	0.84 ± 0.02 (0.46-1)	0.98 ± 0.01 (0.79-1)	**0.90 ± 0.01 (0.65-1)**	0.88 ± 0.02 (0.41-1)	0.88 ± 0.02 (0.54-1)	0.98 ± 0.01 (0.79-1)	**0.93 ± 0.01 (0.76-1)**
Fear	0.96 ± 0.01 (0.75-1)	0.91 ± 0.01 (0.50-1)	0.95 ± 0.01 (0.71-1)	0.94 ± 0.01 (0.74-1)	0.94 ± 0.01 (0.62-1)	0.92 ± 0.01 (0.66-1)	0.95 ± 0.01 (0.46-1)	0.94 ± 0.01 (0.71-1)
Happiness	0.95 ± 0.01 (0.69-1)	0.91 ± 0.02 (0.19-1)	0.68 ± 0.04 (0-1)	0.85 ± 0.02 (0.31-1)	0.95 ± 0.01 (0.69-1)	0.89 ± 0.02 (0.44-1)	0.66 ± 0.04 (0-1)	0.83 ± 0.02 (0.54-1)
Sadness	0.92 ± 0.01 (0.66-1)	0.92 ± 0.01 (0.50-1)	0.97 ± 0.01 (0.66-1)	**0.94 ± 0.01 (0.76-1)**	0.91 ± 0.01 (0.66-1)	0.89 ± 0.01 (0.54-1)	0.96 ± 0.01 (0.58-1)	**0.92 ± 0.01 (0.72-1)**

Dispersion measures include standard error of the mean (SEM) and range. Highlighted in bold font: accuracy for angry items was higher under active tVNS compared with sham, whereas the opposite pattern was observed for sadness.

The critical four-way interaction between type of stimuli, emotion, item difficulty, and tVNS session did not reach significance, neither did all the remaining statistical comparisons (Table 3).

3.5. **HRV and emotion recognition**

As a secondary hypothesis, we attempted to test the idea that efferent baseline vagal tone, as indexed by resting-state HRV, might influence the effectiveness of tVNS in improving the ability to recognize emotions, put forward by Sellaro et al. (2018). We re-ran the above analyses, including mean-centered RMSSD (i.e., the most widely used measure of HRV), averaged over the two sessions, as a covariate.

Including RMSSD as covariate did not change the previous pattern of outcomes, although a new significant source of variance emerged. We observed a significant interaction between item difficulty, tVNS session, and RMSSD (*F*(1,58) = 5.18, *p* = 0.03 , η^2^_p_ = 0.08, MSE = 0.07). Pearson bivariate correlations showed a significant negative relationship between accuracy in active versus sham sessions for easy items and RMSSD (*r* = −0.25, *p* = 0.05), whereas no correlation was observed for difficult items (*r* = 0.12, *p* = 0.34). As recommended by Laborde et al. (2017), we repeated the ANCOVA introducing mean-centered NN50 and HF to check whether the results were confirmed across the main variables reflecting vagal tone. No significant interactions emerged between any of the factors and NN50 (all *ps* ≥ 0.17) or HF (all *ps* ≥ 0.12). Moreover, the main interaction between emotion × session remained significant when independently introducing the HRV covariates [RMSSD: (*F*(2.44,141.38) = 4.48, *p* < 0.01, η^2^_p_ = 0.07, MSE = 0.10); NN50: (*F*(2.44, 141.43) = 4.49, *p* < 0.01, η^2^_p_ = 0.07, MSE = 0.10); HF (*F*(2.42,140.42)=4.52, *p* < 0.01, η^2^_p_ =.07, MSE = 0.10)]. Taken together, we can conclude that HRV did not modulate the effects of tVNS in emotion recognition.

## Discussion

The purpose of the current study was to investigate whether tVNS would enhance the ability to recognize emotional expressions in moving, as opposed to static, bodies. Recent findings suggest a causal role for the vagus nerve in recognizing emotions in the eye region (Colzato et al., 2017) and the face, but not in static bodies (Sellaro et al., 2018). Moreover, findings demonstrate a distinction in the processing of static vs. dynamic emotions (Braddick, 1992; Oram & Perrett, 1994) and the ability to recognize emotions in bodies also has been proposed to be more optimal when a combination of static and dynamic information can be inferred (Atkinson et al., 2004). Because that it also has been suggested the vagus nerve, specifically the ventral complex, may be involved especially in processing dynamic stimuli (Porges, 2001, 2003, 2007, 2011), we hypothesized tVNS to enhance the recognition of emotions in moving, but not static bodies.

Our findings only partially confirmed our hypotheses: the effectivity of tVNS was independent of whether individuals were displayed static or moving bodies, and the direction of the effect depended on the nature of the emotion that was displayed. That is, active compared with sham stimulation improved the ability to recognize anger but decreased the accurate recognition of sadness. No effects of tVNS were found for happiness and fear. Before we discuss these findings in more detail below, we should point out that the proportion of variance in the data explained by the interaction between stimulation and emotion was rather low; thus, our conclusions warrant further investigation and should be taken cautionarily—pending replicability of the observed effects. The low observed effect size may be due to limited variance in the data, given that the average proportion of accurate responses was used as the dependent variable. That is, these proportions theoretically vary between 0 and 1, but the limited number of trials per emotion (i.e., 10 PLDs, 10 FLDs, and 20 static bodies) combined with the overall close-to-ceiling performance (albeit not the case for the recognition of happiness) resulted in a negatively skewed distribution, narrowing the to-be-explained variance. Furthermore, no differences were observed between moving (PLDs or FLDs) and static bodies. A note to be made regarding this evaluation is that analyses contained an unbalanced representation of moving versus static tasks; two of the three tasks (i.e., the PLD and FLD task) were about evaluating moving bodies. Simultaneously, the static bodies task consisted of 80 trials, whereas the PLD and FLD tasks included both 40 trials. We encourage future studies to address these limitations to replicate and confirm the effect of tVNS in recognizing emotions from moving versus static bodies.

Following the suggestion by Sellaro et al. (2018), we additionally investigated whether efferent vagal tone, indexed by resting-state vagally-mediated HRV, influences the effect of tVNS. We found that RMSSD did not change the outcomes but did interact with tVNS and difficulty. Somewhat in line with findings by Kogan et al. (2014), additional analyses yielded a negative correlation of the difference between sham and active tVNS and RMSSD, suggesting that higher RMSSD was related to smaller differences between sham and active tVNS, but only for easy, and not difficult, trials.

The former finding of tVNS improving the ability to recognize anger might be explained based on the Polyvagal theory and associated hierarchical response strategy (Porges, 2001; 2009). This states that adaptive functions and behavioral strategies depend on activation of three autonomic hierarchical subsystems, which provide adaptive responses to life-threatening, dangerous, and safe stimuli, respectively. The most primitive subsystem depends on the dorsal, or unmyelinated vagus, and is associated to immobilization (i.e., freeze, feign death). The next hierarchical subsystem is dependent on the sympathetic nervous system and is associated to mobilizing responses (i.e., fight, flight). The last, phylogenetically newest, subsystem is activated when the environment is perceived as safe and depends on the myelinated, ventral branch of the vagus (i.e., the ventral vagal complex, VVC). It serves, amongst others, social engagement and supports calm behavioral states by inhibiting sympathetic activation. Hierarchy within these three systems is established top-down in a way that phylogenetically newer systems inhibit older subsystems. Hence, only when safety is not perceived or higher systems (i.e., calmness and social engagement) do not lead to adaptive responses, lower subsystems (i.e., fight or flight, eventually possibly freeze) are activated (Porges, 2009). Related to our findings, in contrast to happiness, sadness, and fear, anger expressed by someone else can form a threat that may signal dangerous behavior toward the person observing the emotion. Crucially, the stimuli that we presented were pictures or movies of actors expressing anger. These stimuli, and the context in which they are presented, are inherently safe. Following the idea of hierarchical vagal response strategies (Porges, 2001; 2009), an inability to recognize these stimuli as safe may result in activating a defensive fight-or-flight strategy that is costly and unnecessary in this context (Porges, 2001). Hence, tVNS may have enhanced the identification of the angry stimuli, because enhancing activity of the VVC enables an individual to identify inherently safe stimuli correctly: in this case, a *picture or movie* of an angry person, not a direct threat.

In a similar vein, the decreased ability to recognize sadness induced by active tVNS could be explained by Porges’ Polyvagal hierarchical response theory. Sadness is commonly known as adaptive in a way that it allows one to conserve energy after a loss (Wolpert, 2008). Related to the three hierarchical autonomic subsystems as discussed above, energy conservation (i.e., behavioral shutdown as mentioned by Porges, 2009) is actually associated with decreased activity of the VVC and instead related to the primitive, dorsal vagus (Porges, 2003, 2007, 2009, 2011). If one assumes the hierarchical activation of subsystems (Porges, 2009), then activation of the VVC suppresses the dorsal vagus. If, in addition, we assume a form of perspective taking or affective empathy is needed to correctly identify sadness, increasing activity of the VVC—automatically suppressing activity of the dorsal vagus—should indeed decrease the ability to empathize with sadness, hence recognize it.

Furthermore, the decreased ability to recognize sadness as a result of active tVNS replicates findings by Koenig et al. (2019), who reported that active tVNS decreased recognition of facial displays of sadness in patients suffering major depressive disorder. These findings are in line with the idea that tVNS can be used to enhance mood and treat depressive symptoms (Kong et al., 2018; Liu et al., 2016; Rong et al., 2016). However, our participants did not report high scores on negative affect (and in any case, much higher positive affect). Although we did not assess depressive symptoms, we did screen for (history of) depression diagnosis and made sure participants showed normal empathy baseline levels and no autistic traits. Hence, we argue that our participants should be considered healthy (i.e., nondepressed). Yet, like Sellaro et al. (2018), Koenig et al. (2019) found emotion recognition generally improved in healthy controls. These contradictions with our findings may be explained by our focus on static and moving bodily displays of emotion, instead of facial stimuli. For example, Actis-Grosso et al. (2015) found that, in order to recognize sadness, healthy participants rely more on static cues conveyed by emotional faces, such as those presented in the studies by Sellaro et al. (2018) and Koenig et al. (2019), than on dynamic cues conveyed by emotional bodies. Different stimuli (facial vs. bodily and static vs. moving) may result in different effects and may additionally differ for depressed patients. It is worth noting that cognitive changes (i.e., emotion recognition) precede self-reported changes in mood and subjective depressive symptoms (see Garratt et al., 2007 for a review), and healthy populations differ substantially from patients regarding such processes. That is, mood-enhancing effects, if any, may not become apparent when studying healthy populations (see Sellaro et al., 2015). Hence, our findings warrant further comparison of tVNS in depressive patients versus controls and recognition of facial versus bodily emotional stimuli.

Regarding the lack of effect of tVNS in recognizing fear, it is first important to note that participants performed close to ceiling in recognition, leaving very little room for improvement following tVNS. Nevertheless, speculating on the involvement of the vagus nerve in recognizing fear, the same explanation(s) for the effects of anger and sadness may explain the absence of an effect regarding fear. That is, fear expressed by another person may signal a threat in the environment but only upon sufficient perspective taking, as it does not directly form a threat to the observer. And even less in the current context, where the stimuli (i.e., a picture or movie) are actually safe. Moreover, enhancing the VVC inhibits the autonomic system involved in fight-flight responses by ensuring safety and supporting calmness, which may hence lead to less ability to identify, subsequently empathize with and recognize fear. We can only speculate at this point; more research is needed to disentangle and test these opposing hypotheses.

At this point, what could be the neural mechanisms corroborating the effects of tVNS in modulating emotion recognition? Following the neurovisceral integration model (Thayer & Lane, 2000; 2009), one may consider it likely that the observed effects are due to (stimulation of) the vagus nerve affecting functionality of the CAN. The critical involvement of the CAN as a whole, rather than separate structures, may actually account for the absence of an interaction between HRV and stimulation, given that the former reflects efferent, and the latter afferent, signals of a complex regulatory network of brain structures. Speculating on the role of specific CAN structures, the influence of tVNS on emotion recognition may be explained by many possible affected processes. For example, tVNS may have improved emotion recognition by acting on the dorsolateral–posterior prefrontal–and inferior parietal cortices, which have been found involved in directing attention to the stimulus and holding in mind the goal (Ochsner et al., 2012). An additional possibility is that it improved participant’s ability to use semantic memory to select the goal-appropriate response by supporting activity in ventrolateral prefrontal regions (for a review see Ochsner et al., 2012). This is supported by Kraus et al. (2007), who reported the amygdala and hippocampus are activated by short periods of tVNS. Indeed, to perform an emotion-recognition task, participants need to retrieve previously stored experiences of others’ mental states and their associated bodily expressions in order to compare them to the particular item presented—functions well-known to be related to activity of the hippocampus and amygdala (Hassert et al., 2004; Peelen, Atkinson, Andersson, & Vuilleumier, 2007; Peña et al., 2014). Accordingly, modulation of neural activation in the amygdala and hippocampus could be regarded as a possible working mechanism for the memory-enhancing effects of tVNS on emotion-recognition performance. Nevertheless, such separate structural effects would not account for the differences between emotions (i.e., differential effects for anger and sadness, as observed in the current study), which suggests the effect of tVNS is likely dependent on a complex interaction of neural structures.

The current findings are subjective to a number of limitations and considerations. First, given the high accuracy obtained in this study, future investigations should include response deadlines, which might help to detect more differential effects of tVNS stimulation on emotion recognition. Moreover, to confirm the effect of tVNS in recognizing specifically anger and sadness from bodies, future studies should attempt to replicate this finding while comparing to neutral expressions as well. Second, although it is unlikely that the conscious awareness of the type of stimulation explains the full range of our results, some of our participants correctly reported receiving active stimulation, which may have impacted their performance. Third, although Sellaro et al. (2018) reported no gender effects, perhaps due to not having enough statistical power given their small sample size, it is well-known that gender differences in the ability to empathize and recognize emotions exist. In addition, Williams et al. (2018) showed a moderating role of gender in the relationship between HRV-inferred vagal tone and emotion regulation, possibly generalizing to emotion recognition. As such, it would be interesting for future studies to evaluate the possibility that tVNS differentially affects males and females. Moreover, the somatic marker hypothesis (SMH) (Damasio et al., 1991; Damasio et al., 1996) states that responses to stimuli are affected by somatic markers (i.e., somatic signals associated with emotions that influence decision-making) resulting from bioregulatory processes associated to emotion, in which the afferent vagus provides a key pathway. We did not evaluate the effect of subjective mood state of the participants in the current study, but adapting the perspective of the SMH, it is possible that tVNS enhances signaling of somatic markers (Steenbergen et al., 2020), which would increase the influence of one’s mood state in responding to, for example, the stimuli presented in the current study, a phenomenon also known as emotion egocentricity bias (see also Silani et al., 2013). Furthermore, the current and aforementioned studies evaluated the effect of tVNS on the ability to recognize facial and bodily expressions, but always independently from each other. Bodily expressions have been found to affect the perception of facial expression when conflicting information is presented. Hence, the question remains how the vagal nerve relates to the perception of congruent and incongruent compound stimuli (see also Sellaro et al., 2018). Finally, it would be interesting to understand how the vagus nerve might be causally involved in social approach-avoidance motivation, which requires emotion recognition and seems to be associated with cardiac vagal tone (Movius & Allen, 2005). It is possible that tVNS influences automatic action tendencies in the social domain by affecting approach-avoidance effects in tasks requiring participants to approach or avoid visually presented emotional (happy and angry) faces or bodies.

## Conclusions

Our study supports a possible causal role for the vagus nerve in the ability to recognize emotions expressed in bodies, with differential involvement depending on the specific emotion. In addition to previous findings (Colzato et al., 2017; Sellaro et al., 2018), this contributes to the idea that tVNS may be used to enhance affective processing and social functioning in pathologies possibly related to dysfunctioning of the vagus (i.e., autism, see also Jin & Kong, 2017). More research is needed to evaluate the potential thereof in clinical populations.
